# Assessment of self-medication practices in the context of the COVID-19 outbreak in Togo

**DOI:** 10.1186/s12889-020-10145-1

**Published:** 2021-01-06

**Authors:** Arnold J. Sadio, Fifonsi A. Gbeasor-Komlanvi, Rodion Y. Konu, Akila W. Bakoubayi, Martin K. Tchankoni, Alexandra M. Bitty-Anderson, Iris M. Gomez, Claudia P. Denadou, Joël Anani, Harold R. Kouanfack, Innocent K. Kpeto, Mounerou Salou, Didier K. Ekouevi

**Affiliations:** 1 Département de Santé Publique, Université de Lomé, Faculté des Sciences de la Santé, Lomé, Togo; 2 Centre Africain de Recherche en Epidémiologie et en Santé Publique (CARESP), Lomé, Togo; 3 Programme PACCI – Site ANRS Côte d’Ivoire, CHU de Treichville, Abidjan, Côte d’Ivoire; 4 Conseil Scientifique pour la riposte à la pandémie Covid-19, Lomé, Togo; 5 Laboratoire de Biologie Moléculaire et d’Immunologie, Département des Sciences Fondamentales, Université de Lomé, Lomé, Togo; 6 Université de Bordeaux, INSERM U1219 Bordeaux Population Health Research, ISPED, Bordeaux, France

**Keywords:** COVID-19, *Lomé*-Togo, Prevention, SARS-CoV-2, Self-medication, Traditional medicine

## Abstract

**Background:**

To date, there is no effective treatment for COVID-19, which is a pandemic disease, caused by a novel coronavirus called SARS-CoV-2. In Togo, where four in five people practice self-medication, the absence of a cure for COVID-19 and the constant progression of the disease requires an assessment of self-medication patterns in the context of the pandemic. This study aimed to estimate the prevalence of self-medication to prevent COVID-19 and its associated factors in *Lomé*, Togo.

**Methods:**

A cross-sectional study was conducted in *Lomé*, the capital city of Togo, from April 23rd to May 8th, 2020, with a sample of participants from five sectors: the healthcare, air transport, police, road transport and informal sectors. The participants were invited to provide information about their self-medication practices to prevent COVID-19 in the 2 weeks preceding the survey.

**Results:**

A total of 955 participants (71.6% men) with a median age of 36 (IQR 32–43) were included. Approximately 22.1% were in the air transport sector, 20.5% were in the police sector, and 38.7% were in the health sector. The overall prevalence of self-medication to prevent COVID-19 was 34.2% (95% CI: 31.2–37.3%). The most commonly used products were vitamin C (27.6%) and traditional medicine (10.2%). Only 2.0% of participants reported using chloroquine/hydroxychloroquine. Female sex (aOR=1.90; *p*< 0.001), work in the health sector (aOR=1.89; *p*= 0.001), secondary education level (aOR= 2.28; *p*= 0.043) and university education level (aOR= 5.11; *p*< 0.001) were associated with self-medication.

**Conclusion:**

One-third of the individuals in high-risk populations in *Lomé* practiced self-medication. Intensifying awareness campaigns is crucial to fight misinformation about alleged COVID-19 prevention products on social media.

**Supplementary Information:**

The online version contains supplementary material available at 10.1186/s12889-020-10145-1.

## Background

On January 30th^,^ 2020, the World Health Organization (WHO) declared a public health emergency of international concern due to the advent in China of a disease called COVID-19 caused by a novel coronavirus, SARS-CoV-2, and its rapid spread [1]. Approximately 6 months later, almost 20 million cases and approximately 700,000 deaths have been reported worldwide [2].

To date, there is still no treatment or vaccine for this pandemic. Several studies have evaluated the efficacy of hydroxychloroquine-based treatment with or without azithromycin [3–5]. However, the efficacy of these medicines has not been proven for curative treatment of the disease. Chloroquine and hydroxychloroquine were also evaluated for prophylaxis against COVID-19 in clinical trials among close contacts of individuals diagnosed with COVID-19 and health care workers. Although the preclinical results are promising, there is currently no evidence of the effectiveness of chloroquine/hydroxychloroquine in the prevention of COVID-19 [6].

The COVID-19 epidemic created widespread psychosis and anxiety among the population in sub-Saharan Africa [7]. This could be linked, on the one hand, to the high mortality observed in some countries, such as Italy and Spain, and on the other hand, to the lack of technical resources to combat the disease in sub-Saharan Africa. Regarding the African continent, the WHO indicated that it fears the worst, as the better resourced health-care systems of developed countries have faced enormous difficulties in dealing with the epidemic [8]. Faced with this situation and the variety of information circulating on social media, many plants and substances without the minimum requirements of efficacy and tolerance have been proposed to treat or prevent COVID-19 [9]. The use of these substances without medical advice is considered self-medication, which is defined as taking medicines, herbs or home remedies on one’s own initiative or on the advice of another person without consulting a medical doctor [10]. In the context of the COVID-19 pandemic, cases of poisoning and death have been reported in the USA and Nigeria in persons self-medicating with chloroquine [11, 12].

Health literacy plays an important role in self-medication behavior [13]. Concerning the COVID-19 pandemic and other coronaviruses, the level of knowledge is globally low according to a meta-analysis of 70 scientific articles. Indeed, the proportion of people with a low level of knowledge ranged from 4.3 to 57.9% among health professionals and from 4.0 to 82.5% in the rest of the population [14].

Togo, similar to most West African countries, is experiencing significant population growth (2.8% per year): its population has more than tripled in less than 30 years, rising from 2.7 million inhabitants in 1981 to 8,608,444 inhabitants in 2020 [15]. The demographic context is characterized by (i) a predominantly young population (60% of Togolese are under 25 years old); (ii) a high population density in the coastal regions; and (iii) rapid and uncontrolled urbanization, especially in *Lomé* (capital) [15]. Economically, the gross domestic product per Togolese was 682 U.S. dollars in 2019, making Togo the 11th poorest country in the world [16].

In Togo, although the dispensing of psychoactive drugs is regulated by law and requires a prescription, this is not the case for other drugs, including antibiotics, which can be sold without a prescription [17, 18]. Despite the efforts of the Togolese National Order of Pharmacists to curb the overuse and limit access to antibiotics without a prescription, these drugs are still widely consumed through self-medication [19].

Togo reported its first case of COVID-19 on March 5th^,^ 2020, and the number of cases multiplied by ten in 3 months, with 98 cases and 6 deaths on April 26th [20] compared to 908 cases and 18 deaths on July 31st, 2020 [2]. On October 27, 2020, the country reported 264 cumulative cases and 6 deaths per million inhabitants [21]. The absence of a recognized treatment for the disease and its constant progression requires a re-evaluation of self-medication practices in Togo, where 80% of people resort to self-medication [22] and 60% resort to traditional pharmacopoeia [23]. Thus, this study was conducted to estimate the prevalence of preventive self-medication and its associated factors in an epidemic context where there is no preventive or curative treatment.

## Methods

### Study design and sampling

This study was part of a survey aimed at describing the prevalence of SARS-CoV-2 in high-risk populations in *Lomé* (the capital city of Togo) [24]. This was a cross-sectional study conducted from April 23rd to May 8th, 2020. A total of 955 participants were included, and the prevalence of SARS-CoV-2 was 0.7% according to polymerase chain reaction tests [24].

Participants were recruited from five professional sectors: the healthcare (doctors, nurses, pharmacy auxiliaries, and hospital administrators), air transport, police, road transport (taxi and moto-taxi drivers) and informal (market sellers and craftsmen) sectors. These groups were targeted because they are at high risk of infection during epidemics [25, 26]. Thus, these working professionals had a high probability of being in close contact with travellers or with COVID-19 patients. Participants were eligible to participate in the study if the following four criteria were met: (i) aged ≥ 18 years; (ii) worked in one of the five sectors under study; (iii) were regularly present at their workstation in the month prior to the survey, i.e., had not taken any time off work in the last 30 days and had no sick leave; and (iv) lived in *Lomé* for the past 3 months.

Several sampling methods were used for participant selection based on the expected total size of the target population and the availability of a sampling frame. First, exhaustive recruitment (consisting of the inclusion of all staff present at the moment of the survey) was performed among police (road safety officers) and air transport professionals (*International Airport Gnassingbe Eyadema, Lomé, Togo*) [24]. Second, participants from the informal sector were recruited by open invitation. Third, random sampling (two or three stages) was performed to recruit taxi and moto-taxi drivers (road transport) and health care workers [24]. For example, for the selection of moto-taxi drivers, we performed a two-stage sampling, selecting the company and then the drivers working in the company.

### Sample size

The sample size was calculated using a single proportion population formula with a 95% confidence level. We hypothesized that 50% of the population would practice self-medication, with a 5% margin error. The estimated minimum sample size was 384 participants. A 10% nonresponse rate was anticipated, and the minimum number of participants was estimated at 422 [24]. With a sample size of 955, we reached a 3% margin error.

### Data collection

After the eligibility screening and the participants’ provision of written informed consent, sociodemographic characteristics, COVID-19 epidemiological data and self-medication practices were collected using a standardized questionnaire developed for this survey by a multidisciplinary team involving two medical epidemiologists, two virologists, one pharmacist and one sociologist. All sections of the questionnaire (sociodemographic characteristics; knowledge, attitudes and practices related to COVID-19; symptoms of COVID-19 and biological tests) were developed based on the data reported in the literature since the beginning of the COVID-19 pandemic. The questionnaire was tested on a sample of six medical doctors (those assumed to have the highest level of understanding) and six taxi drivers (those assumed to have the lowest level of understanding), which allowed us to reformulate or remove some questions that seemed complicated or difficult to answer. Five trained medical doctors assisted by students at the end of their medical training administered the questionnaire during a face-to-face interview. The participants were invited to give information on self-medication practices to prevent COVID-19 within the 2 weeks preceding the interview.

### Measurements

Self-medication practice was the outcome variable. It was assessed based on the participants’ selection and use of medicines/drugs alleged to treat or prevent COVID-19 without a physician order in the past 2 weeks.

### Statistical analysis

Descriptive statistics were calculated, and the results are presented as frequencies and percentages for the categorical variables. Quantitative variables are presented as medians and interquartile ranges (IQRs). The prevalence of self-medication was estimated with a 95% confidence interval (95% CI).

Univariable and multivariable logistic model regression were performed to assess factors associated with self-medication with the aim of preventing COVID-19. In the model building, characteristics that had a *p*-value < 0.20 in univariable analysis were considered for the full multivariable models, which were subsequently finalized using a stepwise, backward elimination approach (p-value < 0.05). This procedure allowed the estimation of adjusted odds ratios (aORs) with 95% confidence intervals. Predictor variables were selected as those found to be relevant according to the literature review. The data analyses were performed using R© version 3.4.3 software and the level of significance was set at 5%.

### Ethical considerations

Ethical approval was obtained from the ‘Comité de Bioéthique de Recherche en Santé’ (Bioethics Committee for Health Research) from the Togo Ministry of Health (No. 004/2020/CBRS). Potential participants were informed about the study purpose and procedures, potential risks and protections. Those willing to participate were invited to sign a consent form prior to participation.

## Results

This study was a part of a survey on the prevalence of SARS-CoV-2 in populations at high risk of infection in *Lomé*. A total of 976 people were approached; 21 people refused to be sampled for SARS-CoV-2 and were therefore excluded from the study, for a response rate of 97.8%.

### Sociodemographic characteristics

In total, 955 people with a median age of 36 (IQR 32–43) were included in the study, and 71.6% (*n*=684) were men. Among the recruited participants, 38.7% (*n*=370) were in the health sector, 22.1% (*n*=212) were in the air transport sector, 20.5% (*n*=196) were in the police sector, 12.8% (*n*=122) were in the road transport sector, and 5.8% (*n*=55) were in the informal economy sector. None of the participants had been previously diagnosed as positive for COVID-19 or had been hospitalized in the last 30 days before their enrollment. Almost all of the participants (98.0%; *n*=936) were Togolese. Two-thirds of the participants (66.6%; *n*=636) were living with someone as a couple, and half (51.0%; *n*=487) of them had a university degree. The sociodemographic characteristics by professional sector are summarized in Table 1.

**Table 1:** Sociodemographic characteristics according to sector of activity, Lomé, Togo

	Health	Air transport	Police	Road transport	Informal	Total	p
	***n***=370	***n***=212	***n***=196	***n***=122	***n***=55	***N***=955	
**Age, n (%)**							0.078*
< 50	327 (88.4)	178 (84.0)	180 (91.8)	101 (82.8)	48 (87.3)	834 (87.3)	
≥50	43 (11.6)	34 (16.0)	16 (8.2)	21 (17.2)	7 (12.7)	121 (12.7)	
**Sex, n (%)**							< 0.001**
Men	181 (48.9)	179 (84.4)	168 (85.7)	122 (100.0)	34 (61.8)	684 (71.6)	
Women	189 (51.1)	33 (15.6)	28 (14.3)	0 (0.0)	21 (38.2)	271 (28.4)	
**Nationality, n (%)**							0.018**
Togolese	364 (98.4)	205 (96.7)	196 (100.0)	119 (97.5)	52 (94.5)	936 (98.0)	
Others	6 (1.6)	7 (3.3)	0 (0.0)	3 (2.5)	3 (5.5)	19 (2.0)	
**In couple, n (%)**							< 0.001*
No	169 (45.7)	49 (23.1)	49 (25.0)	42 (34.4)	10 (18.2)	319 (33.4)	
Yes	201 (54.3)	163 (76.9)	147 (75.0)	80 (65.6)	45 (81.8)	636 (66.6)	
**Education level, n (%)**							< 0.001**
None	5 (1.4)	3 (1.4)	0 (0.0)	11 (9.0)	5 (9.1)	24 (2.5)	
Primary	11 (3.0)	2 (0.9)	1 (0.5)	26 (21.3)	4 (7.3)	44 (4.6)	
Secondary	69 (18.6)	105 (49.5)	143 (73.0)	68 (55.7)	15 (27.3)	400 (41.9)	
University	285 (77.0)	102 (48.1)	52 (26.5)	17 (13.9)	31 (56.4)	487 (51.0)	

* Chi square **Fisher exact test

### Products used for self-medication

Table 2 describes the products used for the prevention of COVID-19 by professional sector. The most commonly used products were vitamin C (27.6%) and traditional medicine (10.2%). Chloroquine/hydroxychloroquine was used by 2.0% of the sample, and azithromycin was used by 1.2%.

**Table 2:** Self-medication’s drugs to prevent the infection to SARS-CoV-2 according to sector of activity, Lomé, Togo

	Health	Air transport	Police	Road transport	Informal	Total	p
	***n***=370	***n***=212	***n***=196	***n***=122	***n***=55	***N***=955	
**Chloroquine/Hydroxychloroquine, n (%)**	3 (0.8)	4 (1.9)	4 (2.0)	4 (3.3)	4 (7.3)	19 (2.0)	0.021**
**Azithromycin, n (%)**	5 (1.4)	2 (0.9)	2 (1.0)	1 (0.8)	1 (1.8)	11 (1.2)	0.966**
**Traditional medicine, n (%)**	38 (10.3)	19 (9.0)	17 (8.7)	12 (9.8)	11 (20.0)	97 (10.2)	0.155*
**Vitamin C, n (%)**	181 (48.9)	32 (15.1)	34 (17.3)	8 (6.6)	9 (16.4)	264 (27.6)	< 0.001*

* Chi square **Fisher exact test

### Prevalence of self-medication

The overall prevalence of the use of at least one product to prevent COVID-19 was 34.2% (95% CI: 31.2–37.3%). The prevalence ranged from 16.4% (95% CI= [9.8–23.0]) for participants from the road transport sector to 51.9% (95% CI= [46.8–57.0]) for those from the health sector. Table 3 reports the overall self-medication prevalence by professional sector.

**Table 3:** Overall prevalence of self-medication^a^ to prevent the infection to SARS-CoV-2 according to sector of activity, Lomé, Togo

	n	N	Prevalence (%)	95CI%
**Health**	192	370	51.9	[46.8–57.0]
**Informal**	18	55	32.7	[20.3–45.1]
**Police**	49	196	25.0	[18.9–31.1]
**Air transport**	48	212	22.6	[17.0–28.3]
**Road transport**	20	122	16.4	[9.8–23.0]
**Total**	327	955	34.2	[31.2–37.3]

^a^At least one of: Chloroquine/Hydroxychloroquine, Azithromycin, Traditional medicine, Vitamin C; 95%CI: 95% confidence interval

### Factors associated with the self-medication used

**Fig. 1:**
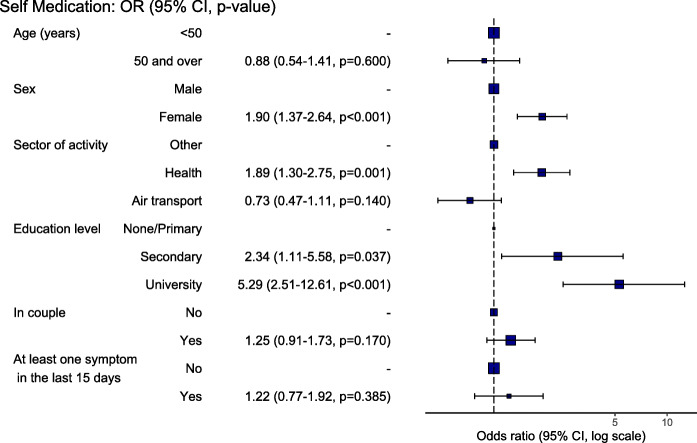
shows factors associated with self-medication to prevent the infection to SARS-CoV-2 in high-risk populations, *Lomé*, Togo in a binary logistic multivariable model. These associations were expressed as adjusted odds ratios. Self-medication was coded as a binary variable (=1 if intake of at least one product and = 0 if not)

In the multivariable logistic regression model, after adjustment for the other variables, three factors were positively associated with self-medication: being female (aOR=1.90; *p*< 0.001), working in the health sector (aOR=1.89; *p*= 0.001) and having attained a secondary or higher education level (aOR= 2.28; *p*= 0.043 for secondary level and aOR=5.11; p< 0.001 for university level). Having at least one symptom related to SARS-CoV-2 was not associated with self-medication (Fig. 1).

## Discussion

The WHO does not recommend self-medication with any medicines, including antibiotics, as a form of prevention or management of COVID-19. Despite the advice of clinicians and governments, 34.2% of the people surveyed in our study used a treatment without a prescription. These treatments included modern treatments as well as traditional medicine. The prevalence of self-medication found in our study is probably related to i) the long delay in finding an appropriate treatment for COVID-19 based on an adequately powered randomized trial [27]; ii) the influence of social media that proposes any type of product to prevent or treat COVID-19 [9]; iii) the influence of leaders (political and religious) who have claimed the efficacy of certain products or who claim to have discovered traditional remedies [28, 29]; and iv) the stigmatization of people infected with SARS-CoV-2, which encourages some people to take care of themselves at home [30].

Chloroquine/hydroxychloroquine was used by 2.0% of the population, and this proportion varied from 0.8% in people working in the health sector and 7.3% in people working in the informal sector. The use of chloroquine/hydroxychloroquine could be linked to the fact that a study published in March 2020 concluded that hydroxychloroquine was effective for the reduction of viral load and recovery time in Covid-19 patients [5]. However, there have been many warnings about the improper use of chloroquine/hydroxychloroquine outside of hospital or clinical trial settings for COVID-19. Its use may increase the risk for arrhythmias or death [31, 32]. Political leaders such as president Trump also claimed to use chloroquine for COVID-19 prevention [28]. This type of declaration shared with the community could be destructive and nonproductive in regard to public health communication.

In our study, azithromycin was used by 1.2% of the sample. Self-medication with antibiotics such as azithromycin could cause harm to the patient and increase the risk of antimicrobial resistance [33]. The low prevalence of the use of azithromycin could be explained by its relatively high cost and by the fact that, in recent years, the Togolese pharmacists’ association has insisted that these products be sold only with a prescription, even the necessity of a prescription is not regulated by law.

While chloroquine and hydroxychloroquine are controlled medicines that are sold in pharmacies, this is not the case for vitamin C. In our study, vitamin C was used by approximately one-third (27.6%) of the participants. Several studies have suggested the effectiveness of a high dose of vitamin C in the management of COVID-19 [34, 35]. However, it is also important to note that high doses of vitamin C may cause side effects, most specifically an increased risk of kidney stones [36].

In April 2020, a traditional medicine called *Covid-Organics* for the prevention and treatment of COVID-19 was promoted in Madagascar [28]. However, the number of cases of COVID-19 in Madagascar quadrupled from 2214 to 10,748 in July 2020 [2, 37]. Several reasons could explain the increase in the number of cases of COVID-19, but this increase raises questions on the effectiveness of *Covid-Organics*, which has not yet been properly tested in therapeutic trials. Artemisia plant, the main component of *Covid-Organics*, has shown some beneficial effects in the treatment of malaria but has not been found to be as beneficial as artemisinin-based combination therapies (ACTs) [38]. The WHO, fearing the risk of the development of a resistance to ACTs linked to the use of this plant, does not recommend it for the treatment of malaria [38]. Furthermore, no studies have proven the efficacy for the prevention or treatment of COVID-19. In our study, one out of ten (10.2%) participants declared that they used traditional medicine for COVID-19 prevention. This finding could be explained by the fact that the use of traditional medicine is common in African culture and relatively less expensive than modern medicines [23], although the composition of these mixtures is usually unknown [23, 39]. In regard to traditional medicine, the WHO welcomes innovations around the world, including repurposing drugs and traditional medicines and developing new therapies in the search for potential treatments for COVID-19 [9]. The WHO is working with research institutions to select traditional medicine products that can be investigated for clinical efficacy and safety for COVID-19 treatment [9].

In this study, self-medication was found to be significantly associated with being female, working in the health sector and having a high school education level or higher. There are conflicting data on the relationship between sex and self-medication [40, 41]. Some studies conducted on self-medication reported that female sex was significantly associated with self-medication. A study conducted among undergraduate students of a private university in Nigeria showed that 88.2% of females versus 71.1% of males reported using self-medication [42]. In Spain, the prevalence of self-medication was 16.93% (2715) for women and 14.46% (1469) for men (*p*< 0.05) in a study about sex differences in self-medication [43]. The reason for the association between female sex and self-medication is not clearly known, but in the context of the COVID-19 outbreak, greater anxiety among women, as described in Iran and Italy, cannot be excluded [44, 45].

A 2018 systematic review and meta-analysis of observational studies conducted in Ethiopia showed that healthcare professionals and students were the main practitioners of self-medication [46]. In our study, self-medication was associated with working in the health sector. Knowledge and access to prescription-only medicines are potential factors of self-medication among health professionals. A lack of time to consult with a doctor and the desire to keep one’s health status secret were also mentioned as factors that could explain self-medication among health care personnel [47]. According to the WHO, approximately 10% of all COVID-19 cases globally are among health workers. In Africa, information on health workers’ infections is still limited, but preliminary data show that health worker cases make up more than 5% of cases in 14 countries in sub-Saharan Africa alone, and in four of these countries, infections among health workers constitute more than 10% of all infections [48]. The higher risk of infection among health care professionals, their knowledge of drugs and their ease of access to drugs may also explain their higher practice of self-medication [49, 50].

Self-medication has often been associated with a lower education level. A study on knowledge and self-medication with antibiotics conducted in a Lebanese adult sample reported that self-medication was significantly associated with low education level (*p*=0.036) [51]. This was not the case for the present study conducted in the context of the COVID-19 outbreak. Indeed, participants with a high school level or higher were more likely to self-medicate. This finding could be explained by the fact that a good knowledge of diseases is known to be associated with self-medication [52–54]. The greater access of the educated population to the internet and their ability to understand information about treatment (which is often published in official languages) found on social networks may also explain this trend.

Surprisingly, a history of clinical manifestation was not associated with self-medication in our study, which confirmed the finding that self-medication was more likely to be used for the prevention of COVID-19 and not to treat specific clinical manifestations of COVID-19, which are similar to malaria symptoms.

This study has some limitations. We did not collect data on the doses of the drugs used and the length of time they were used. For traditional medicines, the composition of the different traditional products used was not collected. It should also be noted that in the Togolese context, traditional medicines are very often used in combination with modern medicines. Another limitation of this study is that the questionnaire used was developed entirely by our team and had never been used before. Even if the questionnaire had been pretested, biases (primacy effect, order effect, etc.) could not be excluded. Furthermore, the study was based on declarative data, which may have led to an underestimation of the prevalence of self-medication due to social desirability bias. Finally, according to the characteristics of the surveyed population (people with a high risk of SARS-CoV-2 infection), the extrapolation of these results to the general population should be performed with great caution.

## Conclusion

This study was the first to assess the prevalence of self-medication to prevent COVID-19 in Togo. Approximately one-third of people reported ever having performed self-medication. Vitamin C and traditional medicines were the most commonly used products. Health professionals, women and people with a high level of education were the most likely to practice self-medication. Close collaboration is needed with pharmacists so that they will not sell chloroquine/hydroxychloroquine without a medical prescription. It is also important to fight against misinformation about supposed COVID-19 preventive products on social media with campaigns to improve awareness. Psychology is also important in COVID-19 response strategies, as reducing anxiety and ambient psychosis could reduce the use of dangerous self-medication. Studies in the general population should be conducted to confirm these results.

## Supplementary Information

**Additional file 1. **Surveillance Survey of SARS-CoV-2 Infection in Populations at Risk of *Lomé-Commune* in 2020: Survey sheet. This is a questionnaire developed for data collection for this study. The file is made up of two parts, an information and contact details part and a survey form part.

## Data Availability

The datasets used and/or analysed during the current study are available from the corresponding author upon reasonable request.

## Abbreviations

95%CI: 95% confidence interval
aOR: Adjusted Odds Ratio
Covid-19: Coronavirus Disease
IQR: Interquartile range
OR: Odds Ratio
SARS-CoV-2: Severe acute respiratory syndrome coronavirus 2
WHO: Wold Health Organization
