# Supplementary material for: Assessment of self-medication practices in the context of the COVID-19 outbreak in Togo
Source: BMC Public Health. 2021 Jan 6;21:58. doi: 10.1186/s12889-020-10145-1 (PMC7787400; doi:10.1186/s12889-020-10145-1)
Supplement: Supplementary file 1 — Additional file 1. Surveillance Survey of SARS-CoV-2 Infection in Populations at Risk of Lomé-Commune in 2020: Survey sheet. This is a questionnaire developed for data collection for this study. The file is made up of two parts, an information and contact details part and a survey form part. [file 12889_2020_10145_MOESM1_ESM.pdf]

Information and contact details

First name: ……

Last Name: ………...

Phone number 1: ……………………………………………………………………………………………………….

Phone number 2: ……………………………………………………………………………………………………….

Address: ……….

……..

Laboratory ID: I_S_I_R_I_A_I_S_I____I____I____I ____I

Participant ID: I____I____I____I____I____I____I____I____I____I____I____I____I

(Participant ID: First 3 letters Last name then first 3 letters First name, Sex (M/F), Year of birth)

NB: The Participant ID must be identical to the one reported on the survey form

Contact persons

First name: ……………………………………………

Last name: ……………………………………………

Family Relationship: …………………………………

Phone number: ……………………………

First name: ……………………………………………

Last name: …………………………………………

Family Relationship: …………………………………

Phone number: …………………………

<u>Important:</u> This sheet "Information and contact details" is to be detached from the survey sheet after completion and to be kept under lock and key!

Survey Sheet

Inclusion date: I ____ I ____ I / I ____ I ____ I / I____I____I____I____I

Laboratory ID: I_S_I_A_I_R_I_S_I____I____I____I ____I

Participant ID: I____I____I____I____I____I____I____I____I____I____I____I

(Participant ID: First 3 letters Last name then first 3 letters First name, Sex (M/F), Year of birth)

Inclusion place: ……………………………………………………………………………………………………….

Investigator ID: ____

1. Sociodemographic characteristics

Q101. Age (years): I___I___I

Q102. Gender:❑ Male¹ ❑ Female²

Q103. Nationality:❑ Togolese¹ ❑ Others² (Specify): ……………………………………………………… ..

Q104. Profession:❑ Airport professionals¹ ❑ Customs² ❑ Taxi driver³ ❑ Moto taxi driver⁴ ❑ Health professional⁵

❑ Merchants⁶ ❑ Patient⁷ ❑ Hotel professional⁸ ❑ Other⁹: ………………………………………

If health professional

Q104.1. Function:❑ Physician¹ ❑ Medical assistant² ❑ Nurse³ ❑ Midwife⁴ ❑ Pharmacy auxiliaries⁵

❑ Other⁹: ……………………………………………………………………………….

Q104.2. Exercice place : ❑ CHU Campus¹ ❑ *Clinique Biasa*² ❑ *Clinique l'espérance*³ ❑ *Clinique Autel d'Elie*⁴

❑ *CHU Sylvanus Olympio*⁵ ❑ Others⁶ (Specify): ………………………….

Q105. Marital status :❑ Single¹ ❑ Married² ❑ Divorced³ ❑ Widowed⁴

Q106. Study level: ❑ Out of school⁰ ❑ Primary¹ ❑ Secondary² ❑ Superior³

2. Epidemiological link

Q201. Concept of travel outside Togo in the last two months:❑ Yes¹ ❑ No⁰

Q201.1. If yes, date of return to Togo: I ____ I ____ I / I ____ I ____ I / I____I____I____I____I

Q202. Concept of contact with an infected person:❑ Yes¹ ❑ No⁰

Q202.1. If Yes, link with this person: …………………………………………………………….

Q203. BCG vaccination status: ❑ Vaccinated¹ ❑ Not vaccinated⁰ ❑ Don't know⁹⁹

Q204. How many people have you had contact with for 30 minutes or more in the past 48 hours? I____I____I

Q205. How many people have you had contact with for 30 minutes or more in the past 48 hours without barrier measures? I____I____I

Q206. How many people do you live with in your household? I____I____I

Q207. How many people do you live with in your room? I____I____I

3. Knowledge, Attitudes and Practices towards COVID-19

Q301. Have you ever heard of COVID-19? ❑ Yes¹ ❑ No⁰

Q301.1. If yes, how? ❑ Media¹ ❑ Word of mouth² ❑ Others³ (Specify): ……………………

Q302. A person with the virus can infect others even if they do not have symptoms: ❑ True¹ ❑ False²

Q302. Concept of preventive self-medication against COVID-19

Q302.1. Taking Chloroquine / Hydroxychloroquine? ❑ Yes¹ ❑ No⁰

Q302.2. Taking azithromycin? ❑ Yes¹ ❑ No⁰

Q302.3. Taking traditional medicine preparation? :❑ Yes¹ ❑ No⁰

Q302.4. Daily vitamin C intake? ❑ Yes¹ ❑ No⁰

Q303. Protective measures used: ❑ Social distancing¹ ❑ Hand hygiene²

❑ Wearing a fabric mask³ ❑ Wearing a surgical mask⁴

❑ FFP⁵ mask port ❑ No protection⁰

4. Symptoms (If applicable)

Q401. Have you had in the past 15 days or are you currently experiencing any of the following signs?

❑ None ⁰ ❑ Fever¹ ❑ Headache² ❑ Joint / muscle pain (body aches) ³ ❑ Sore throat⁴

❑ Rhinorrhea⁵ ❑ Breathing difficulties (shortness of breath) ⁶ ❑ Anosmia (difficulty perceiving odors) ⁷

❑ Ageusia (difficulty perceiving the taste of food) ⁸ ❑ Abdominal pain⁹ ❑ Diarrhea¹⁰ ❑ Cough¹¹

❑ Unusual fatigue¹² ❑ Others (Specify) ¹³: ___________________

Q402. Start date of symptoms (DD-MM-YYYY): I ____ I ____ I- I ____ I ____ I- I____I____I____I____I

Q403. Temperature today (Degrees Celsius): I____I____I. I____I

Q404. Have you contacted a health professional since the onset of symptoms?❑ Yes¹ ❑ No⁰

Q404.1. If yes to question 404, how? ❑ By calling "111" ¹ ❑ By calling my attending physician²

❑ By going to a health center³

5. Biological examination

Q501. Date of collection (DD-MM-YYYY): I ____ I ____ I- I ____ I ____ I- I____I____I____I____I

Q501.1. PCR result:❑ Negative⁰ ❑ Positive¹ ❑ Doubtful²

Q501.2. RDT results:❑ Absence of antibodies⁰ ❑ IgG¹ ❑ IgM²
